# Sex-specific roles of hippocampal microRNAs in stress vulnerability and resilience

**DOI:** 10.1038/s41398-022-02267-4

**Published:** 2022-12-06

**Authors:** Maayan Krispil-Alon, Vladimir Jovasevic, Jelena Radulovic, Gal Richter-Levin

**Affiliations:** 1 Sagol Department of Neurobiology, University of Haifa, Haifa, Israel; 2 The Integrated Brain and Behavior Research Center (IBBR), University of Haifa, Haifa, Israel; 3 Psychology Department, University of Haifa, Haifa, Israel; 4 Department of Pharmacology, Northwestern University, Feinberg School of Medicine, Chicago, IL 60611 USA; 5 Dominick P. Purpura Department of Neuroscience, Albert Einstein College of Medicine, Bronx, New York, NY 10461 USA

**Keywords:** Epigenetics and behaviour, Psychiatric disorders

## Abstract

Contrary to intuition, most individuals are resilient to psychological trauma and only a minority is vulnerable. Men and women are known to respond differently to trauma exposure, however, mechanisms underlying the relationship between sex differences and trauma resilience and vulnerability are not yet fully understood. Taking advantage of the Behavioral Profiling approach, which enables differentiating between ‘affected’ and ‘unaffected’ individuals, we examined sex-associated differences in stress exposure effects on hippocampal expression of selected stress-related GABA-A receptor targeting miRNAs. Levels of the miRNA-144 and miRNA-33 were measured in male and female affected (vulnerable, e.g., higher freezing time) and unaffected (resilient) rats. In male rats, increased levels of miRNA-144 and miRNA-33 were observed in the dorsal dentate gyrus (dDG) and ventral dentate gyrus (vDG) respectively, of stress-exposed but unaffected animals. In females, we observed an increased expression of miRNA-144 and miRNA-33 in the ventral cornu ammonis 1 (vCA1) of affected animals. Accordingly, we inhibited miRNAs expression selectively in hippocampal subregions using oligonucleotides containing locked nucleic acid bases, to examine the miRNAs’ causal contribution to either vulnerability or resilience to stress in each sex. Inhibition of miRNA-144 in dDG and miRNA-33 in vDG in males resulted in an increased prevalence of vulnerable animals, while inhibition of miRNA-144 and miRNA-33 in vCA1 in females increased the proportion of resilient animals. The current findings reveal a critical sex-associated difference in the role of miRNAs in stress vulnerability and resilience. This novel understanding of sex-associated epigenetic involvement in the mechanism of stress-related psychopathologies may help improve gender-specific diagnosis and effective treatment.

## Introduction

Sex differences in stress responses have been consistently demonstrated in human and animal models [1–4]. For instance, men are more likely to turn to substance abuse following a stressful event [5], while women are more likely to suffer from anxiety and trauma-related disorders [6–8]. Furthermore, there are non-sex related individual differences. Studies have shown that the majority of people are resilient to stress, and a relatively small proportion is vulnerable and may develop long-term stress-related psychopathologies [9, 10]. The underlying mechanisms by which individual and sex differences influence stress resilience and vulnerability are still largely unknown and are of fundamental importance to improving treatment of stress-related psychopathologies. It is possible that these mechanisms involve epigenetics, and specifically alterations in microRNAs (miRNAs) expression [11–14]. Accumulating evidence shows that dysregulation of miRNAs in body fluids and brain tissues may be associated with stress-related conditions in both patients and animal models [15–20].

Several in vivo studies indicate that miRNAs could be involved in vulnerability and resilience to stress-related mental disorders [21–23], as well as in stress-related sex differences [24, 25]. However, the contribution of miRNA to the mechanism of sex-related individual patterns of trauma response, was not yet systematically studied. Some miRNAs were demonstrated to contribute to the post-transcriptional regulation of gene expression of GABA-A receptors, following stress exposure [26]. For instance, exposure of mice to acute adult stress led to differential expressions of several GABA-A receptor targeting miRNAs in the hippocampus. Among them are miRNA-144 and miRNA-33 which target four or more mRNAs encoding GABA-A receptors [27]. A pilot study showed that similar changes to these miRNAs occurred in rats’ hippocampus following juvenile stress exposure (Fig. S2). Changes in miRNAs following stress exposure were not yet, to the best of our knowledge, studied in different subregions of the hippocampus.

Different hippocampal subregions are suggested to have distinct functions. While the dorsal hippocampus was found to be involved in cognition and spatial mapping, the ventral hippocampus is regarded as playing a role in stress responses, emotional memory formation and anxiety [28, 29]. Additionally, differences were found between the involvement of the dentate gyrus (DG) and cornu ammonis 1 (CA1), with regards to sensitivity and impact of exposure to stress [30, 31]. Having found hippocampal alterations in miRNA-144 and miRNA-33 following stress exposure, it would be interesting to pin down site-specific GABA-A related miRNA expression in the aforementioned subregions of the hippocampus and the extent to which sex influences the vulnerable or resilient response patterns.

Therefore, the first aim of the current study was to explore whether there is a difference in the expression pattern of the GABA-A receptor targeting miRNA-144 and miRNA-33 transcripts in the dorsal and ventral dentate gyrus (dDG, vDG, receptively) and dorsal and ventral CA1 (dCA1, vCA1, receptively) hippocampal areas, in the stress exposed affected rats compared to that of the stress exposed but unaffected, male and female rats. In the second part, aiming to directly examine the contribution of miRNA-144 and miRNA-33 to trauma vulnerability and resilience in males and females, we inhibited their functioning in the relevant subregions of the hippocampus.

## Material and methods

### Animals

For experiment 1, male and female Sprague–Dawley rats (postnatal day (PND) 22 on arrival, weight 30–50 g, Harlan Laboratories, Jerusalem, Israel), were group housed (22 ± 2 °C; light–dark cycle: 12/12 h), with water and food ad libitum. For experiment 2, male rats arrived on PND 45 (weight 175–199 g) and female rats arrived on PND 22 (weight 30–50 g) and were kept in the same conditions. Animals were randomly allocated to experimental groups. Sample size for each section was based on extensive previous experience [3, 4, 32–39]. Experimenters were blind to the group allocation during behavioral test performance. Animal care and experiments were performed in accordance with the NIH Guide for care and use of laboratory animals and approved by the University of Haifa ethical committee (610/19).

### Experimental procedure

**Fig. 1:**
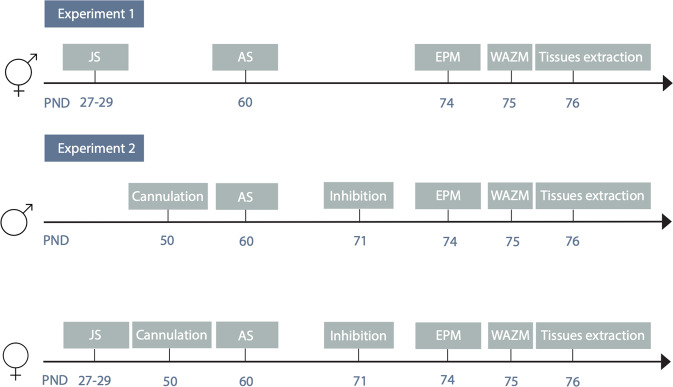
Timeline of experiment 1 and experiment 2 experimental procedure.

The experimental design is depicted in Fig. 1. Rats were assigned to two experimental paradigms:

#### Experiment 1

Following arrival and acclimation all rats were randomly assigned to one of four cohorts: Control (male: *n* = 20, female: *n* = 17); juvenile stress (JS, male: *n* = 23, female: *n* = 20); adult stress (AS, male: *n* = 23, female: *n* = 20); and combined JS + AS (male: *n* = 23, female: *n* = 20). JS and JS + AS rats were subjected to JS protocol. In adulthood, all rats were habituated to the water-associated zero maze (WAZM), and following this, only groups AS and JS + AS were immediately subjected to underwater trauma (UWT) stress. 14 days later, all rats were re-exposed to the elevated plus maze (EPM) and WAZM for the purpose of behavioral profiling. 24 h later, tissue samples were extracted and assessment of miRNA-144 and miRNA-33 levels in the different subregions of the hippocamps was conducted. In total, this experiment was replicated four times.

#### Experiment 2

miRNA inhibition. Since, based on results in experiment 1, we hypothesized that knocking down miRNA-144 and miRNA-33 would compromise resilience in males, we used a protocol with a relatively low baseline prevalence of affected animals, to more effectively detect a potential increase in the proportion of affected animals by our manipulation [32]. Locked nucleic acid (LNA) inhibition was performed for miRNA-144 in the dDG and for miRNA-33 in the vDG following exposure to UWT (see Fig. S6 for cannula placement, and additional supporting data in table S1 and Figs. S4 and S5). Male rats were randomly assigned to one of four cohorts: exposure to adult stress and LNA inhibition (AS-LNA: *n* = 7); exposure to adult stress without inhibition (AS-SCR: *n* = 10); LNA inhibition without stress exposure (CTR-LNA: *n* = 8); and no stress exposure/no inhibition (CTR-SCR: *n* = 7).

In females, since, based on results from experiment 1, we hypothesized that blocking the functioning of the miRNAS will support stress resilience, we employed the combined exposure to JS and UWT, a stress protocol with an observed initial high baseline prevalence of affected animals, in order to more effectively be able to detect an increase in the proportion of unaffected animals. Rats were randomly assigned to one of four cohorts: exposure to juvenile and adult stress and LNA inhibition (JS + AS-LNA: *n* = 8); exposure to juvenile and adult stress without inhibition (JS + AS-SCR: *n* = 10); LNA inhibition without stress exposure (CTR-LNA: *n* = 13); and no stress exposer and no inhibition (CTR-SCR: *n* = 10). LNA inhibition was performed for miRNA-144 and miRNA-33 in the vCA1 following exposure to the UWT.

Fourteen days later all rats were re-exposed to the EPM and WAZM and were subjected to behavioral profiling. In total, this experiment was replicated four times.

### Juvenile stress (JS) exposure

JS rats were exposed to 3 different stressors on 3 consecutive days under full light condition (as described before [3, 33]): PND 27–15 min of forced swim stress (water temperature 22 ± 2 °C); PND 28 - elevated platform (12 × 12 cm, 70 cm above floor level) exposure for 3 × 30 min (1 h ITI in the home cage); PND 29—2 h restraint.

### Water-associated zero maze (WAZM)

The WAZM is an integrated wet and dry context maze that enables an association of the maze with an UWT [34]. On PND 60, following 5 min habituation to the room, rats were habituated to the WAZM for additional 5 min, on the dry arena, facing the closed part of the WAZM, in one of its open arms. Then rates were removed from the dry platform and placed in the aquatic center of the apparatus, where they were subjected to UWT stress for 45 s, using a special metal net (AS). Following the procedure, the rats were dried and returned to their home cages. For the test, following 2 min habituation to the room, rats were placed on the dry arena at the same location. Rats were given 5 min to explore the arena, during which their behavior was recorded and analyzed by EthoVision XT10 video tracking system (Noldus, Wageningen, Netherlands) (For an elaborate description of this procedure see [35]).

### Elevated plus maze (EPM)

For a different context measurement of emotional behavior not directly associated with the trauma, elevated plus maze (EPM) test was carried out on PND 74, 24 h before the WAZM test, as described before [40]. After 2 min habituation to the room, rats were placed in the center of the maze (110 × 110 cm, 70 cm above the floor; two opposing open arms/closed arms), facing an open arm and were allowed to freely explore the arena maze for 5 min. Behavior was recorded and analyzed by EthoVision XT10 video tracking system (Noldus, Wageningen, Netherlands).

### Behavioral profiling

Analysis of individual behavior, indicating resilience or vulnerability, was used to establish the different effects of JS and AS conditions, as well as miRNA-144 and miRNA-33 LNA inhibition on rats’ behavioral phenotype. In this analysis the classification of animals’ behavioral profiles was based on the upper or lower 20th percentiles of anxiety-like behaviors (WAZM and EPM tests) of the control group distribution. Classification criteria were based on the lower 20th percentiles of control’s distribution, for time spent in open arms (WAZM and EPM), distance traveled in open arms (WAZM) and total distance traveled (WAZM) and on the upper 80th percentiles for freezing time (WAZM and EPM). All rats were individually discerned for each criterion. For the final classification, every rat that demonstrated a behavioral profile that fell within a minimum of 4 out of 6 criteria was classified as affected [36].

### Surgery and cannulation

The procedures were performed under Ketamine anesthesia (10% Ketamine 100 mg/kg, and 2% dormitor, 10 mg/kg (Vetmarket, Petah-Tikva, Israel), both i.p.). Rats were restrained in a stereotaxic apparatus (Stoelting instruments) and implanted with a stainless-steel guide cannula (23 gauge, thin walled). Double guided cannulas were bilaterally implanted in the dDG (−3.7 mm posterior, ±2.3 mm lateral and −3.8 mm ventral to bregma) and vDG (−5.8 mm posterior, ±4.5 mm lateral and −7.1 mm ventral to bregma) in male, and in the vCA1 (−6 mm posterior, ±5.6 mm lateral and −7.6 mm ventral to bregma) in female. The cannulas were set in place using acrylic dental cement and secured by three skull screws. A stylus was placed in the guide cannula to prevent clogging. For the following first 3 days, rats were treated with analgesics (Dipyrone, 0.3 ml/kg, s.c.; Vetmarket) and antibiotics (15% Amoxycillin, 0.2 ml/kg, s.c.; Vetmarket, Petah Tikva, Israel) and were allowed to recover in their home cage for a total of one week before behavioral testing began. For microinjection, the stylus was removed, and a 14-gauge (for the dDG) or 16-gauge (for the vDG and vCA1) injection cannulae, extending 1.0 mm from the tip of the guide cannula, were inserted. The injection cannula was connected via polyethylene PE20 tubing to a Hamilton microsyringe driven by a microinfusion pump (PHD1000, Harvard Apparatus, USA). Bilateral microinjections of a 0.5 µl volume were delivered over 1 min. The injection cannula was left in each position for an additional 1 min before withdrawal to minimize dragging of the injected liquid along the injection tract [41].

### Quantitative real-time PCR (qRT-PCR) for assessment miRNA expression

Total RNA was extracted using miRNeasy RNA Isolation Mini Kit (Qiagen, Hilden, Germany, Cat. No. 217004). Reverse transcription was carried out using qScript cDNA Synthesis Kit (Quanta Biosciences, Gaithersburg, USA, Cat. No. 95047) following the manufacturer’s protocol. miRNA-144-3p (Quanta Biosciences, Cat. No. MIR SET), miRNA-33-5p (Quanta Biosciences, Cat. No. MIR SET) and miRNA-381-5P (Quanta Biosciences, Cat. No. MIR SET) expression were assessed using SYBR Green qRT-PCR amplification (5 ng total RNA in 20 μl total reaction volume, Cat. No. 95074) using specific primers (0.4 μl each, Quanta Biosciences, Gaithersburg, USA) according to manufacturer’s instructions. Real-time PCR analysis was performed on a QuantStudio 3 real-time PCR system (Thermo Fisher Scientific Inc., Waltham, MA, USA), using the following PCR conditions: (95 °C for 3 min Holding stage, followed by 40 cycles (95 °C for 15 s, 60 °C for 45 s) [42].

For data analysis, the mean cycle threshold (CT) was determined for each triplicate assay and relative quantification of each target gene was conducted with the ddCT method [43], normalizing each sample to the overall content of cDNA using RNU6 as an internal control {dCT; dCT = [CT (target gene)] − [CT (RNU6)]}. Normalization of all ddCT values was done relative to Control group with ddCT = dCT(sample) − mean dCT (Control group). Transformation to RQ values for a specific target gene and area was done according to RQ = 2−ddCT with RQ(Control) = 1.

### miRNA inhibition

miRCURY LNA miRNA144-3P inhibitors (sequence 3′-5′: AGTACATCATCTATACTGT, Qiagen, Hilden, Germany) and miRCURY LNA miRNA33-5p inhibitors (sequence 5′-3′: GCAATGCAACTACAATGCA, Qiagen, Hilden, Germany) were dissolved in water to 400 μM, heated at 65 °C for 10 min, cooled on ice, and diluted in artificial cerebrospinal fluid (ACSF) to a final working concentration of 40 μM. The inhibitors have a full phosphorothioate backbone modification (PS) (added for enhanced stability and potency). Scrambled LNA, which does not bind to any cellular miRNA, were used as a negative control (sequence 5′-3′: TAACACGTCTATACGCCA, Qiagen, Hilden, Germany) [27].

### Statistical analysis

The sample sizes of each experiment were determined based on our previous studies of stress-emulating paradigms [32, 33, 35, 36]. Data were analyzed using the IBM SPSS Statistics Software (IBM, Armonk, NY, USA). For comparing anxiety like behavior (e.g., freezing time in the WAZM) following different stress exposures, a one-way ANOVA followed by Fisher’s LSD post hoc test was applied. The prevalence of affected vs. unaffected populations between the groups in experiment 1 and experiment 2 was compared using Pearson’s chi squared test. For miRNA level differences between type of stress exposures or behavioral profiles, and their interactions, a two-way ANOVA, followed by Fisher’s LSD post hoc test was applied when data was normally distributed. For non-normally distributed data were analyzed by Kruskal-Wallis test followed by pairwise two-sided comparisons. Homogeneity of variance was confirmed with Levene’s test for equality of variances. Significance was accepted when *p* < 0.05. Outliers were defined as two or more standard deviations from the mean and removed from the analysis. GraphPad Prism 7.0 (GraphPad Software, San Diego, CA), and BioRender (https://biorender.com/) were used to create figures.

## Results

### Group and individual differences in response to stress exposure in males and females

**Fig. 2:**
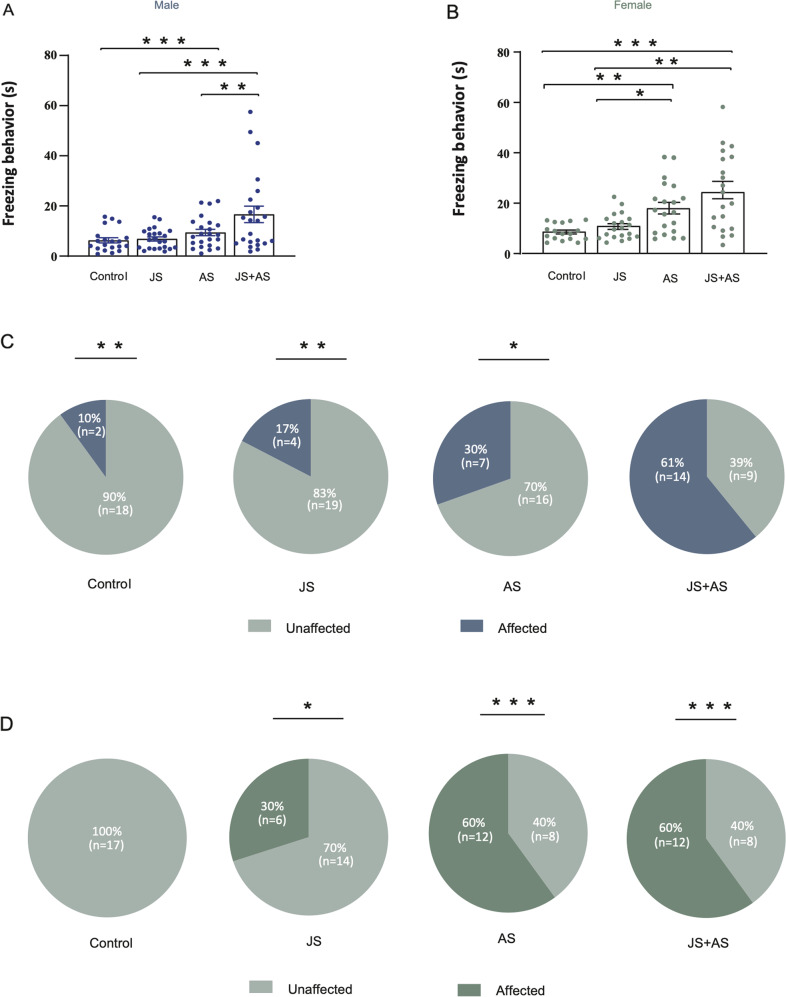
Averaged freezing time group effects and individual behavior profiles following different stress exposures in males and females. **A** In males, the JS + AS group spent more time freezing in the WAZM compared to all other groups. Number of animals: Control: *n* = 20, JS: *n* = 23, AS: *n* = 23, JS + AS = 23. **B** In females, both AS and JS + AS groups showed increased freezing behavior in the WAZM compared to control and JS rats. Number of animals: Control: *n* = 17, JS: *n* = 20, AS: *n* = 20, JS + AS = 20. **C** Behavioral profiling according to anxiety-like behaviors in male rats, revealed an increase in the prevalence of affected animals in the JS + AS group compared to that of all other groups. **D** In females, behavioral profiling analysis uncovered a significantly higher prevalence of affected animals among JS + AS and AS groups equally, compared to that of the control group. A significantly higher proportion of affected animals, though less pronounced, was detected in the JS group compared to that of the control group. **A**, **B** Data presented as individual values, with bars and whiskers representing means and standard errors, respectively. **C**, **D** Values are the % and number of affected and unaffected animals in each group. *P < 0.05, **P < 0.01, ***P < 0.001.

Freezing behavior as expressed in total freezing time in the WAZM was measured in males and females. In males, freezing time of the JS + AS group was significantly longer (Fig. 2A; F_(3,85)_ = 6.19, *p* < 0.001) compared to all other groups (Control: *P* < 0.001, JS: *p* < 0.001, AS: *p* < 0.01). In females, both AS and JS + AS groups showed more freezing behavior (Fig. 2B; H_(3)_ = 19.94, *p* < 0.001) compared to control (AS: *P* < 0.01, JS + AS: *P* < 0.001) and JS groups (AS: *P* < 0.05, JS + AS: *P* < 0.01), demonstrating an overall increase in anxiety-like behaviors after exposure to combined stress in males and to AS and combined stress in females.

Sex differences in response to trauma exposure between groups of males and females have been established in the past [44–46], however, due to the fact that some individuals are more susceptible to stress than others, a comparison of the different groups’ means does not expose differences in stress induced changes at the individual level between males and females. Hence, we used behavioral profiling in order to identify vulnerable and resilient individuals among the male and female groups following exposure to the various stressors.

Behavioral profiling revealed sex-related differences. In males, increased prevalence of affected animals was found in the JS + AS group compared to all other groups (Fig. 2C; Control: χ2 (1) = 11.85, *p* < 0.01; JS: χ2(1) = 9.13, *p* < 0.01; AS: χ2 (1) = 4.29, *p* < 0.05).

In females, increased prevalence of affected rats was found in both the AS and JS + AS groups compared to the control group (Fig. 2D; AS: χ2 (1) = 15.1, *p* < 0.001, JS + AS: χ2 (1) = 15.1, *p* < 0.001, receptively). In addition, though less pronounced, increased prevalence of affected animals was found also in the JS groups compared to the control group (Fig. 2D; JS: χ2 (1) = 6.09, *p* < 0.05).

### miRNA levels in affected and unaffected male rats

**Fig. 3:**
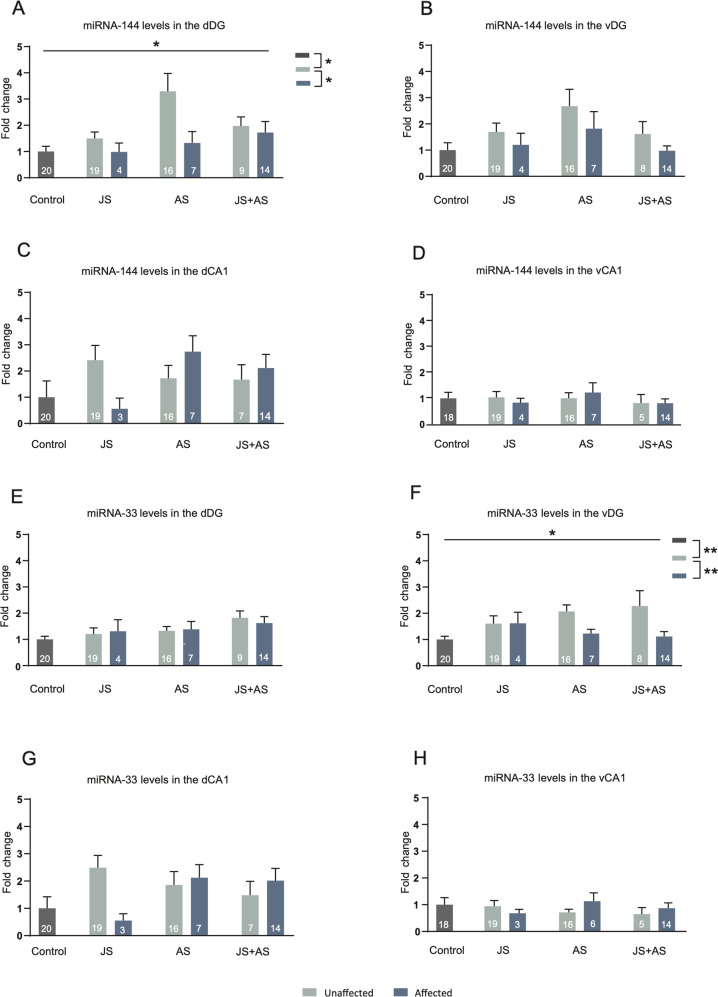
miRNA levels in affected and unaffected male rats. **A** Increase in miRNA-144 levels occurred exclusively in the dorsal dentate gyrus (dDG) of unaffected rats with a history of JS, AS or combined JS + AS, whereas no main effects for stress exposure group or behavioral profile were observed in the ventral dentate gyrus (vDG) (**B**), dorsal Cornu Ammonis 1 (dCA1). **C** or ventral CA1 (vCA1) (**D**). Within the vDG, an increase in miRNA-33 expression was evident in stress-exposed, but unaffected rats (**F**). No main effects for group or profile were observed for miRNA-33 expression in the dDG (**E**) dCA1 (**G**) and vCA1 (**H**). Data presented as means and standard errors of fold-change, relative to control. *Significant difference between profiles, *p* < 0.05, **p < 0.01.

For miRNA-144, a significant main effect was demonstrated for behavioral profile that was restricted to the dDG (Fig. 3A; H_(2)_ = 7.84, *P* < 0.05). Post hoc comparisons revealed increased miRNA-144 levels specifically in unaffected rats compared to those in affected rats (*p* < 0.05) and unexposed control rats (*p* < 0.05). A similar trend, not statistically significant, was observed in the vDG (Fig. 3B; H_(2)_ = 4.43, *P* = 0.11). No significant main effects for behavioral profile or type of stress exposure were observed in either the dCA1 or vCA1 (Fig. 3C, D).

For miRNA-33, a significant main effect for behavioral profile was observed in the vDG (Fig. 3F; F(1,81) = 5.78, *p* < 0.05). Post hoc comparisons revealed a significant increase of miRNA-33 transcript level in stress exposed, unaffected rats compared to that of affected and control rats (*p* < 0.01, *p* < 0.01, receptively). No significant main effects of type of stress exposure or behavioral profile were observed in the dDG (Fig. 3E), dCA1 (Fig. 3G) or vCA1 (Fig. 3H).

For miRNA-381, no significant main effects for behavioral profile or type of stress exposure were observed in either the dDG, vDG or dCA1 (Fig. S3A–C). (Fig. SD) In the vCA1 a significant main effect for type of stress exposure was observed (F_(2,74)_ = 3.65, *p* < 0.05). Post hoc comparisons revealed lower levels of miRNA-381 expression in JS + AS exposed animals compared to the AS group (*p* < 0.05).

### miRNA levels in affected and unaffected female rats

**Fig. 4:**
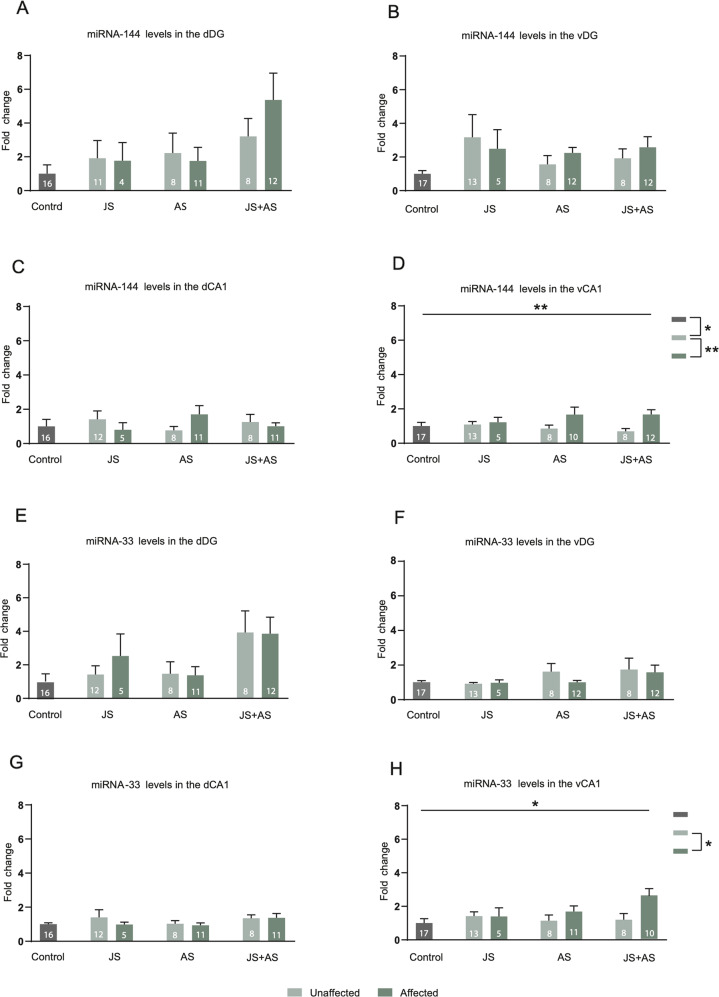
miRNA levels in affected and unaffected female rats. **A**–**C** No main effects or interactions were observed in miRNA-144 levels in the dDG, vDG and dCA1. **D** Increased miRNA-144 levels occurred specifically in the vCA1 of affected compared to unaffected rats. **E**–**G** No main effects or interactions were observed in miRNA-33 levels in the dDG, vDG and dCA1. **H** Within the vCA1 increased miRNA-33 levels occurred in stress affected compared to those in unaffected rats. Data presented as means and standard errors of fold-change relative to control. *Significant difference between profiles, *p* < 0.05.

For miRNA-144, a significant main effect for behavioral profile was observed in the vCA1 (Fig. 4D; F_(1,66)_ = 7.66, *P* < 0.01), Post hoc comparisons revealed increased miRNA-144 levels specifically in affected compared to unaffected rats (*P* < 0.01) and unexposed control rats (*p* < 0.05). No significant main effects for behavioral profile, type of stress exposure or interactions were observed in the dDG, vDG or in the dCA1 (Fig. A–C).

For miRNA-33, a significant main effect for behavioral profile was observed in the vCA1 (Fig. 4H; F_(1,68)_ = 4.13, *P* < 0.05), post hoc comparisons showing increased transcript levels in stress affected compared to those in unaffected rats (*P* < 0.05). No significant differences were observed in the dDG, vDG or in the dCA1 (Fig. 4E–G).

For miRNA-381, no significant main effects for behavioral profile or type of stress exposure were observed in either the dDG, vDG or dCA1 (Fig. S3E–G). (Fig. S3H) In the vCA1 a significant main effect for type of stress exposure was observed (F_(2,67)_ = 5.10, *p* < 0.001). Post hoc comparisons revealed lower levels of miRNA-381 expression in JS + AS exposed animals compared to that in the other groups (control: *p* < 0.01, JS: *p* < 0.001, AS: *p* < 0.001).

### The proportions of affected/unaffected following LNA inhibition

**Fig. 5:**
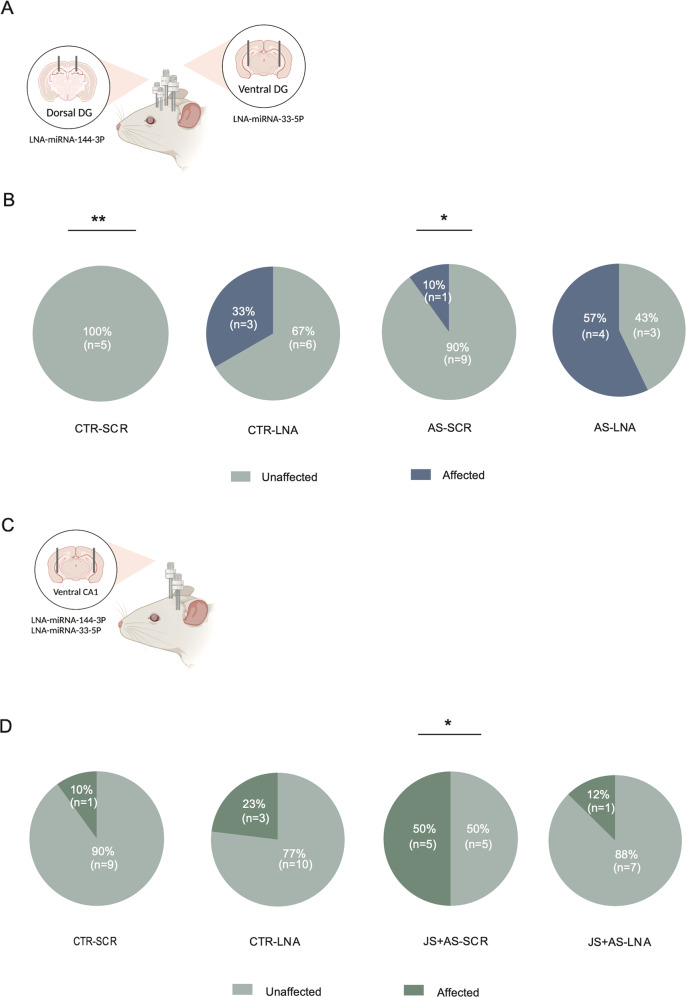
Behavioral profiles following miRNA-144 and miRNA-33 LNA inhibition in males and females. **A** Schematic of cannula implantations in males. **B** In male rats, behavioral profiling revealed an increased prevalence of affected rats in the AS-LNA group compared to that in the AS-CTR group. Number of animals: CTR-SCR: *n* = 7, CTR-LNA: *n* = 8, AS-SCR: *n* = 10, AS-LNA = 7. All values are mean ± SEM. **C** Schematic of cannula implantations in females. **D** In female rats, behavioral profiling revealed an increased prevalence of unaffected rats in the JS + AS-LNA group compared to that of the JS + AS-SCR rats. Number of animals: CTR-SCR: *n* = 10, CTR-LNA: *n* = 13, JS + AS-SCR: *n* = 10, JS + AS-LNA = 8. Values are the % and number of affected and unaffected animals in each group. *Different from JS + AS-SCR, *p* < 0.005.

Next, following the initial findings in males (experiment 1), which demonstrated an association between increased levels of miRNA-144 (dDG) and miRNA-33 (vDG) and resilience, we inhibited these miRNAs following adult stress using LNA inhibition. A causal relation should lead to reduced resilience, and thus to increased proportion of affected subjects. To increase the likelihood of identifying effects of the manipulation, inhibition was conducted following AS exposure, which is expected, without manipulation, to lead to low proportion of affected animals. The results showed that indeed, inhibition of miRNA-144 in the dDG and of miRNA-33 in the vDG in male rats (for cannula location see Fig. 5A), significantly increased the prevalence of affected animals in the AS-LNA group (Fig. 5B; χ2(1) = 4.41, *P* < 0.05) compared to CTR-SCR (Fig. 5B; χ2(1) = 5.6, *P* < 0.01) (Fig. 2C).

In contrast, in females, higher levels of miRNA-144 and miRNA-33 in the vCA1 were found to be associated with increased vulnerability to stress. Inhibiting these miRNAs in females could be thus expected to enhance resilience. Therefore, we examined the impact of inhibition of those miRNAs in vCA1 in animals exposed to JS + AS, a condition which is, by itself, associated with an initial high proportion of affected animals. Indeed, inhibition of miRNA144 and miRNA33 in the vCA1 in female rats (for cannula location see Fig. 5C), significantly increased the proportion of ‘unaffected’ rats in the AS-LNA group compared to that of AS-SCR (Fig. 5D; χ2(1) = 2.81, *P* < 0.05) and that of the control group (Fig. 5D; χ2(1) = 5.6, *P* < 0.05).

## Discussion

The current results are a continuation of studies emphasizing the importance of shifting away from working with group averages and towards analyzing responses to stress and trauma at the individual level [3, 32, 33, 35, 36, 47–49]. The current results demonstrate significant differences in alterations in miRNAs in exposed animals between ‘affected’ and ‘unaffected’ individuals. Such differences would have been completely masked by averaging the results of the exposed groups.

The alterations found in miRNA expression were region-specific. These results join previous findings, emphasizing that the role of expression alterations of specific proteins should be discussed in the context of the specific region, in which they were identified [32, 36, 37]. This, of course, represents a significant challenge for planning translational targeted interventions. However, the first step in any such consideration is to identify region-specific alterations, interventions in which would be sufficient to induce a phenotypic effect at the behavioral level. Here, we could demonstrate that the region-specific alterations in expression of miRNAs identified following trauma exposure were indeed sufficiently involved in vulnerability or resilience, to the level that manipulating the functionality of these alterations was sufficient to alter the outcome of trauma exposure. Differential expression of neuromodulators such as serotonin, norepinephrine or dopamine, or the interneuron-localized neuropeptide Y (NPY), were found to mediate stress resilience and vulnerability in a trauma-exposed population, at the individual level [50–52], and epigenetic mechanisms were suggested to mediate such stress-related individual differences [53, 54].

But results did not only indicate individual differences and region specificity, but also sex-associated differences. Trauma-related psychopathologies, including post-traumatic stress disorder (PTSD) and post-traumatic depression, are more prevalent in women than in men [55–58]. Gonadal hormones such as testosterone and estrogen as well as interacting genetic factors, have long been believed to differentially affect the hypothalamic-pituitary-adrenal (HPA) axis or modulate hippocampal functioning both in humans and in animal models [59–61], potentially affecting the risk of developing pathology directly, or through epigenetic mechanisms [62]. Even though some reports could not demonstrate such association (see, e.g., refs. [63–65]), the majority of studies have demonstrated differences in stress responses between males and females following exposure to traumatic events, and have documented this difference in animal models and experimental tests of stress and trauma [3, 38, 39, 66–69]. In accordance with that, here, we found no difference between males and females in the prevalence of ‘affected’ individuals following exposure to JS + AS, but only in females there was a significant increase in the prevalence of ‘affected’ individuals following exposure to AS alone.

An important focus of the current study was the identification of sex-associated differences in miRNAs expression alterations. Specifically, in males, up-regulation of miRNA-144 in the dDG and miRNA-33 in the vDG, was related to resilience while in females, these miRNAs increased expression in the vCA1 was associated with greater vulnerability (Fig. 3A, F, D). In accordance with that, down-regulation of those miRNAs was sufficient to induce increased vulnerability in males and increased resilience in females. The population of rats profiled as ‘unaffected’ was reduced in males and increased in females following LNA inhibition in the relevant regions (Fig. 5B, D). To the best of our knowledge this is the first study demonstrating enhanced resilience in females following specific miRNA inhibition. Furthermore, alterations in two different miRNAs (males and females), and in two brain regions (males) have led us to employ a dual intervention, handling two miRNAs simultaneously. The success of this approach, as revealed by significant effects on stress vulnerability and resilience, encourages its employment in future studies. Identification of the downstream, possibly also sex-specific targets of miRNA-33 and miRNA-144 will further clarify the mechanisms of their differential effects as they relate to both gender and subregions. However, clearly, these are not the only molecules and the only regions involved. Future studies may investigate the specific target genes associated with these miRNAs. It may also be beneficial to examine hormonal involvement (e.g., corticosterone, estradiol, or progesterone) in sex-related resilience/vulnerability mechanisms.

In summary, the current results reveal critical sex-associated differences in hippocampal epigenetic responses to trauma exposure, which contribute to stress vulnerability in females, and to stress resilience in males. The results emphasize the significant value of dissociating neural mechanisms underlying coping with stress from those mechanisms underlying failure to cope, which could lead to the development of trauma-related psychopathologies. Towards that goal, it is critical to differentiate between ‘affected’ and ‘unaffected’ individuals. The findings here, that resilience is associated with significant alterations in expression of specific miRNAs in specific hippocampal regions, add to a body of findings indicating that stress resilience is not a passive quality but rather an active response which enables coping with the experience. Better understanding of the mechanisms at the basis of such active resilience, may eventually be translated to ways of enhancing stress resilience.

## Supplementary information

Supplementary Material
