# Correction to: Sex-specific roles of hippocampal microRNAs in stress vulnerability and resilience

**DOI:** 10.1038/s41398-023-02333-5

**Published:** 2023-01-31

**Authors:** Maayan Krispil-Alon, Vladimir Jovasevic, Jelena Radulovic, Gal Richter-Levin

**Affiliations:** 1 Sagol Department of Neurobiology, University of Haifa, Haifa, Israel; 2 The Integrated Brain and Behavior Research Center (IBBR), University of Haifa, Haifa, Israel; 3 Psychology Department, University of Haifa, Haifa, Israel; 4 Department of Pharmacology, Northwestern University, Feinberg School of Medicine, Chicago, IL 60611 USA; 5 Dominick P. Purpura Department of Neuroscience, Albert Einstein College of Medicine, Bronx, New York, NY 10461 USA

**Keywords:** Epigenetics and behaviour, Psychiatric disorders

Correction to: *Translational Psychiatry* 10.1038/s41398-022-02267-4, published online 06 December 2022

The original version of this article unfortunately contained a mistake in an author name. Rudulovic, J. should be Radulovic, J. The original article has been corrected.

