# Supplementary material for: Sex-specific roles of hippocampal microRNAs in stress vulnerability and resilience
Source: Transl Psychiatry. 2022 Dec 6;12:503. doi: 10.1038/s41398-022-02267-4 (PMC9726879; doi:10.1038/s41398-022-02267-4)
Supplement: Supplementary file 1 — Supplementary Material [file 41398_2022_2267_MOESM1_ESM.docx]

**Sex-specific roles of hippocampal microRNAs in stress vulnerability and resilience**

Maayan Krispil-Alon^123^ (PhD), Vladimir Jovasevic^5^(PhD), Jelena Rudulovic^4^(MD/PhD), Gal Richter-Levin^123^(PhD)

^1^Sagol Department of Neurobiology, University of Haifa, Israel

^2^The Integrated Brain and Behavior Research Center (IBBR), University of Haifa, Israel ^3^Psychology Department, University of Haifa, Israel

^4^Dominick P. Purpura Department of Neuroscience, Albert Einstein College of Medicine, Bronx, New York, NY 10461, USA

^5^Department of Pharmacology, Northwestern University, Feinberg School of Medicine, Chicago, IL 60611, USA

**Figure S1.** **Stress-induced miRNAs showing up-regulation 2 weeks after exposure to stress.** Stress induced overlapping patterns of miRNA expression (left). Most of the up-regulated miRNAs are predicted or proved to target multiple GABA-A related proteins (right).

A


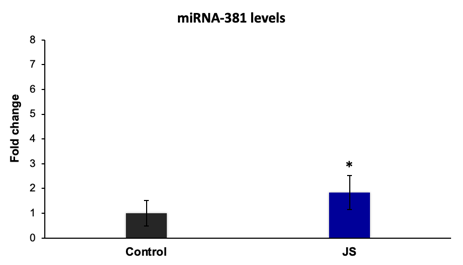

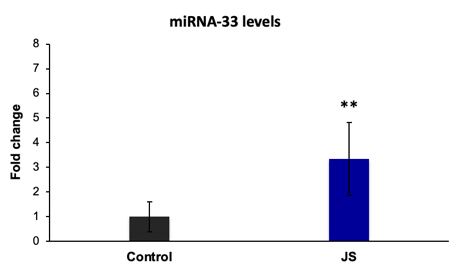

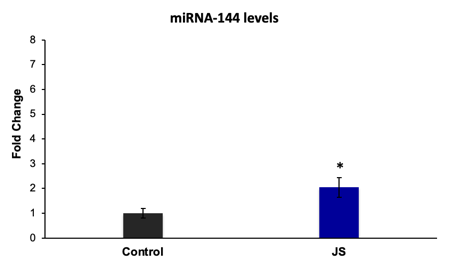


C

B


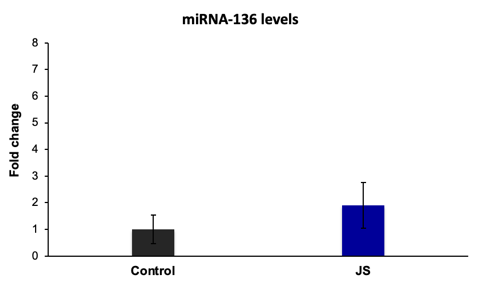

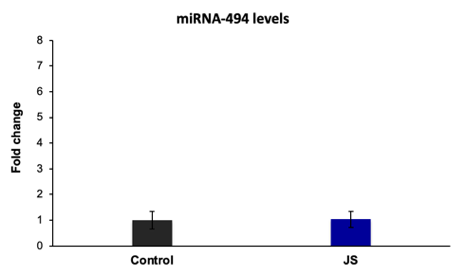


E

D

**Figure S2.** **Results of pilot study following juvenile stress (JS) exposure in rats.** Sustained increase was demonstrated in rat hippocampus in (A) miRNA-144 (t_(24)_ = 4.92, p<0.05), (B) miRNA-33 (t_(24)_=9.34, p<0.01), and (C) miRNA-381(t_(24)_=5.63 p<0.05) targeting GABA-A-related proteins, following JS. No significant increase was found in (D) miRNA-136 and (E) miRNA-494. All samples were normalized to let-7b. The current study investigated potential increased expression of these miRNAs following JS, AS, and JS+AS. Error bars represent mean±s.e.m. *p < .05, ** p < .01.

B

C

A


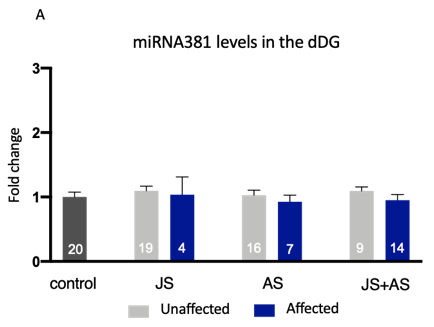

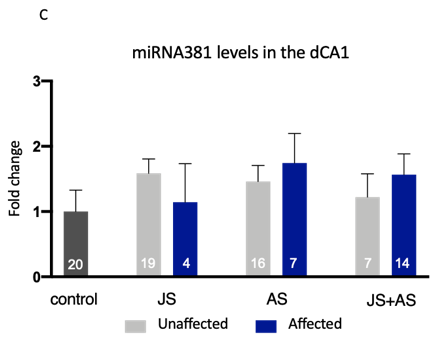

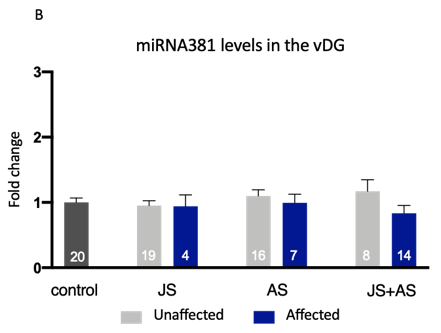


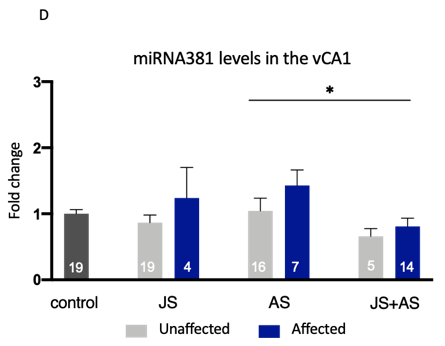


D

G

F

E


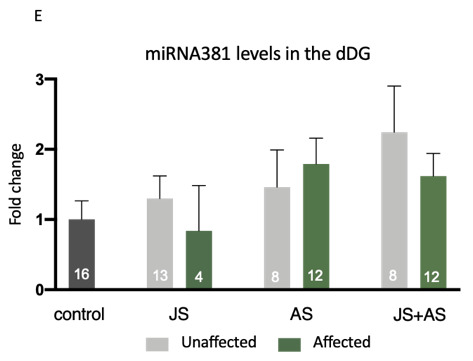

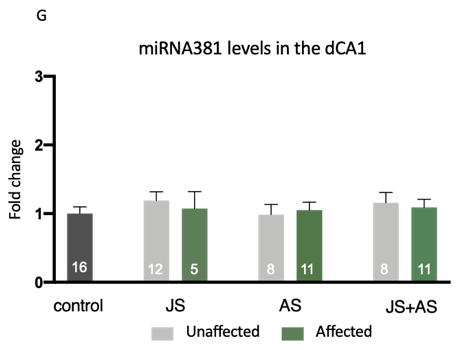

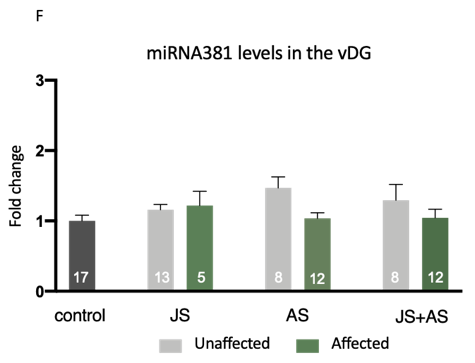


**
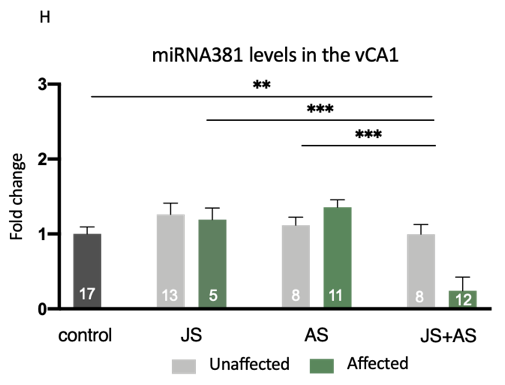
**

H

**Figure S3.** **miRNA-381 levels in male rats**. No significant main effects for behavioral profile or type of stress exposure were observed in either the dDG, vDG or dCA1 (A-C). (D) In the vCA1 a significant main effect for type of stress exposure was observed (F_(2,74)_=3.65, p<0.05). Post hoc comparisons revealed lower levels of miRNA-381 expression in JS+AS exposed animals compared to the AS group (p<0.05). **miRNA-381 levels in female rats.** No significant main effects for behavioral profile or type of stress exposure were observed in either the dDG, vDG or dCA1 (E-G). (H) In the vCA1 a significant main effect for type of stress exposure was observed (F_(2,67)_=9.6, p<0.001). Post hoc comparisons revealed lower levels of miRNA-381 expression in JS+AS exposed animals compared to that in the other groups (control: p<0.01, JS: p<0.001, AS: p<0.001). Data presented as means and standard errors of fold-change, relative to control. *p < .05, ** p < .01, *** p < .001.

Table S1: Predicted target genes of miRNA-144-3P and miRNA-33-5P.

| miRNA-144-3P* | | | | | | | | miRNA-33-5P* | |
| --- | --- | --- | --- | --- | --- | --- | --- | --- | --- |
| \| IGIP \| \| --- \| \| UCHL3 \| \| NFE2L2 \| \| EIF4G2 \| \| RARB \| \| ATP5G2 \| \| ARID2 \| \| TSPAN3 \| \| GPR183 \| \| GBE1 \| \| TFRC \| \| MSX1 \| \| SORCS3 \| \| HERPUD1 \| \| TNFSF11 \| \| UBE2G1 \| \| MEF2A \| \| MEIS2 \| \| VPS4B \| \| KIF2A \| \| PCDH18 \| \| PTHLH \| \| THAP1 \| \| PURA \| \| PANK1 \| \| CCNG2 \| \| ABTB2 \| \| PEX11B \| \| FBXO32 \| \| BRPF1 \| \| CAV2 \| \| RASA1 \| \| GDF10 \| \| APPBP2 \| \| TBX1 \| \| MYCL \| \| MYCN \| \| RGMA \| \| FST \| \| PAFAH1B1 \| \| EPB41L2 \| \| KCNMB2 \| \| TFAP4 \| \| NR2F2 \| \| CASK \| \| UBE2D1 \| \| MOB4 \| \| ACBD3 \| \| PCSK5 \| \| PHF3 \| \| KHDRBS3 \| \| CYTH3 \| \| NUP37 \| \| ST18 \| \| PLA2G4A \| \| E2F8 \| \| GALNT3 \| \| KDM3A \| \| GATA3 \| \| MAP7D1 \| | \| ETS1 \| \| --- \| \| SENP7 \| \| BBC3 \| \| ALDH1A3 \| \| AHDC1 \| \| GSPT1 \| \| MARCKS \| \| KPNA1 \| \| ATP1B1 \| \| MAPK6 \| \| APP \| \| CAPZA1 \| \| USP42 \| \| ZBTB18 \| \| RIN2 \| \| LHX2 \| \| PDE7B \| \| ZDHHC21 \| \| ETV1 \| \| LIMCH1 \| \| CELF2 \| \| SEC24A \| \| FAM222B \| \| EML1 \| \| PPTC7 \| \| SMPD3 \| \| ITSN2 \| \| CCDC85A \| \| SREK1 \| \| ZFX \| \| PTPN12 \| \| GLCCI1 \| \| STRN \| \| NLK \| \| HS2ST1 \| \| SUCLA2 \| \| KLF12 \| \| GRM5 \| \| HTRA3 \| \| RGS17 \| \| FMR1 \| \| NPTX1 \| \| FBN2 \| \| LSM14A \| \| MDM4 \| \| SOX1 \| \| CAMKK2 \| \| TET2 \| \| EBF1 \| \| VKORC1L1 \| \| RAB30 \| \| ALS2 \| \| ACBD5 \| \| YLPM1 \| \| SCN1A \| \| SLC23A2 \| \| DCBLD2 \| \| TMEM65 \| \| USP46 \| \| GCLC \| | \| CBX4 \| \| --- \| \| UCK2 \| \| CDS1 \| \| MBNL1 \| \| ZFP36L2 \| \| NCOA7 \| \| FLRT3 \| \| FAT4 \| \| DGCR2 \| \| MAFK \| \| HOXA10 \| \| SYNCRIP \| \| CDK8 \| \| PDE3B \| \| KHDRBS2 \| \| TRIB1 \| \| NOL4 \| \| AJAP1 \| \| CCNE2 \| \| PTP4A1 \| \| SALL1 \| \| CLK4 \| \| CACNA2D1 \| \| CASC4 \| \| ZBTB34 \| \| ZCCHC2 \| \| RNF111 \| \| FAM126B \| \| RFX3 \| \| FBXW11 \| \| SGPP1 \| \| SS18 \| \| RPGRIP1L \| \| MEGF9 \| \| ELAVL2 \| \| PRICKLE1 \| \| ABHD17B \| \| PHLDA1 \| \| FOS \| \| RNF139 \| \| SP4 \| \| SSX2IP \| \| FAM196A \| \| PTGS2 \| \| ZDHHC17 \| \| TGIF1 \| \| CDH11 \| \| SLC20A2 \| \| CDH20 \| \| EZH2 \| \| MORN4 \| \| NID2 \| \| SON \| \| SCAMP1 \| \| KAT6A \| \| RAB1A \| \| MCF2L \| \| SAP30L \| \| PPP2R2A \| \| CACNB2 \| | \| STAG1 \| \| --- \| \| CDC42EP3 \| \| SLITRK4 \| \| PHTF2 \| \| MGRN1 \| \| EIF5 \| \| ST6GALNAC3 \| \| GJA1 \| \| EHMT1 \| \| FNDC3A \| \| ZEB1 \| \| CPEB2 \| \| SIK1 \| \| ANKRD17 \| \| SRSF10 \| \| LRRTM2 \| \| PIEZO1 \| \| ACSL4 \| \| PLXNC1 \| \| SLC12A2 \| \| JPH1 \| \| PALM2 \| \| STC1 \| \| FAM76B \| \| BACH2 \| \| AMMECR1 \| \| ZC3H12C \| \| RASD2 \| \| ARID4B \| \| HAPLN1 \| \| DR1 \| \| RAPGEF2 \| \| MAP3K4 \| \| PPP1R16B \| \| MYT1 \| \| ZSWIM5 \| \| CAV3 \| \| PPFIA1 \| \| MBNL2 \| \| PPP3R1 \| \| MED14 \| \| POU2F1 \| \| NACC2 \| \| DLG5 \| \| PHF20L1 \| \| RANBP9 \| \| ROCK2 \| \| CAMSAP2 \| \| ATXN1 \| \| PIK3C2A \| \| KCND2 \| \| KPNA3 \| \| TSPAN12 \| \| ARID1A \| \| PPIG \| \| SNN \| \| ZBTB21 \| \| TJP1 \| \| PRRC2C \| \| FMN2 \| | \| EEA1 \| \| --- \| \| NEUROD1 \| \| BBX \| \| UBE2D3 \| \| ZC3H11A \| \| SULT4A1 \| \| CPEB1 \| \| AEBP2 \| \| APLP2 \| \| IPO8 \| \| BEGAIN \| \| NPNT \| \| RAP1A \| \| SLC4A10 \| \| MTX3 \| \| ADAMTSL3 \| \| PPP1R13B \| \| CDYL \| \| KLF6 \| \| ZBTB37 \| \| AGRN \| \| BAZ2B \| \| UBA2 \| \| EPN2 \| \| NEK7 \| \| TGIF2 \| \| PBX3 \| \| ADAMTS17 \| \| ATXN1L \| \| HERC2 \| \| PTPRJ \| \| SHISA6 \| \| OTUD4 \| \| INO80D \| \| PDE4A \| \| CDH5 \| \| ERO1L \| \| PAPOLG \| \| USP47 \| \| ARHGAP26 \| \| IMPACT \| \| CREBZF \| \| BCL2L11 \| \| IRS1 \| \| BTBD3 \| \| NAA30 \| \| KBTBD8 \| \| IFFO2 \| \| ARRDC3 \| \| SH2B3 \| \| MED13 \| \| TSHZ3 \| \| NR3C1 \| \| UBR3 \| \| MBNL3 \| \| ABHD17C \| \| SLC7A11 \| \| ATP2B2 \| \| EFNA5 \| \| RALGPS1 \| \|  \| \|  \| \|  \| | \| PHF6 \| \| --- \| \| ASH1L \| \| RUNX1 \| \| AGO3 \| \| KCNA1 \| \| TRIO \| \| PPP2R5E \| \| ABCA1 \| \| REV3L \| \| TM9SF3 \| \| ARHGEF3 \| \| TANC2 \| \| MEF2D \| \| FAM222A \| \| HIPK3 \| \| PRKAA1 \| \| SEL1L \| \| ZSWIM6 \| \| RXRB \| \| SEPHS1 \| \| PLEKHG1 \| \| SCN8A \| \| MTMR2 \| \| ZFAND3 \| \| NOG \| \| HNRNPF \| \| GOLGA4 \| \| SLC4A4 \| \| SLC1A1 \| \| RAP1B \| \| SLAIN2 \| \| SETD5 \| \| PRKCE \| \| HDAC9 \| \| BCL9 \| \| RORA \| \| TTN \| \| RREB1 \| \| CTDSPL2 \| \| EMP2 \| \| TSPAN5 \| \| NOVA1 \| \| ZEB2 \| \| MARK1 \| \| UBN2 \| \| TOP1 \| \| RAP2C \| \| FRYL \| \| TRAPPC8 \| \| FLRT2 \| \| ANTXR2 \| \| RAP2B \| \| PCDH7 \| \| NR6A1 \| \| PTPN9 \| \| FAM199X \| \| MITF \| \| FBXL3 \| \| PAPPA2 \| \| SIX4 \| | \| ACVR2B \| \| --- \| \| PDS5B \| \| CREBRF \| \| ZFHX4 \| \| MAP3K9 \| \| ASAP1 \| \| ICK \| \| PURB \| \| CCDC88A \| \| SFRP1 \| \| TNRC6A \| \| CEP170 \| \| WDFY3 \| \| ERBB4 \| \| DSTYK \| \| ROBO2 \| \| FBXW7 \| \| LMTK2 \| \| NSD1 \| \| SH3PXD2A \| \| TBL1XR1 \| \| BMPR1B \| \| ADAMTS15 \| \| FGFR1OP \| \| TENM3 \| \| PNRC1 \| \| SLC8A1 \| \| AFAP1 \| \| NACC1 \| \| SMARCA4 \| \| UBE2A \| \| EIF5A2 \| \| FAM168A \| \| FRS2 \| \| LIFR \| \| MED12L \| \| TRIM44 \| \| NR1D2 \| \| SPRED1 \| \| TSC22D2 \| \| BICD2 \| \| DIP2B \| \| TNRC6B \| \| PPFIA4 \| \| IDH2 \| \| ATP2B1 \| \| HNRNPU \| \| MET \| \| GABRB2 \| \| TNRC18 \| \| NRK \| \| SOCS7 \| \| PKNOX1 \| \| FAM84A \| \| KCTD10 \| \| ATAD2B \| \| SMOC1 \| \| ZFHX3 \| \| HEY2 \| \| HAT1 \| | \| ATRX \| \| --- \| \| DMD \| \| MYO1E \| \| EMP1 \| \| DNMT3A \| \| VLDLR \| \| WEE1 \| \| GET4 \| \| USP31 \| \| SIK2 \| \| PLAT \| \| GPR174 \| \| TANC1 \| \| EBPL \| | \| CROT \| \| --- \| \| HMGA2 \| \| EN2 \| \| GRIK2 \| \| CELF2 \| \| YWHAH \| \| STRBP \| \| SLC25A25 \| \| GLCCI1 \| \| EEF1A1 \| \| DCUN1D1 \| \| NAA30 \| \| PIM1 \| \| ANKRD44 \| \| ESCO1 \| \| RGS7BP \| \| HADHB \| \| NIPAL4 \| \| RAP2A \| \| NSF \| \| TBC1D12 \| \| CPT1A \| \| NAA15 \| \| RGMA \| \| BTBD3 \| \| SLC12A5 \| \| ABCA1 \| \| CDK6 \| \| GAS1 \| \| MRPL49 \| \| LRRC4 \| \| ARID5B \| \| TMEM86A \| \| HOXC13 \| \| ZC3H12C \| \| CNTN4 \| \| NPC1 \| \| MSRB3 \| \| SREK1 \| \| PIM3 \| \| NHLH2 \| \| SEC24C \| \| EPHA8 \| \| NDNF \| \| GRM8 \| \| SEMA3A \| \| MTF1 \| \| DNAJB5 \| \| SGIP1 \| \| ZFP36L1 \| \| TTC28 \| \| SATB2 \| \| SMAD7 \| \| BTBD2 \| \| CEND1  SEPT7 \| \| CADPS \| \| DUSP6 \| \| SOCS5 \| \| SYNCRIP \| \| AHCYL1 \| \| CACNA1C \| \| \| SMARCA5 \| \| \| EBF1 \| \| \| MAPK6 \| \| \| AGGF1 \| \| \| KCND3 \| \| \| SPIN1 \| \| | \| CADM2 \| \| --- \| \| FGF14 \| \| ROBO2 \| \| ICK \| \| KPNA4 \| \| PDPK1 \| \| CTNND1 \| \| SH2B3 \| \| MAP3K3 \| \| PDGFRA \| \| CPEB2 \| \| RORA \| \| NFIA \| \| ANKRD12 \| \| DACH1 \| \| PDIK1L \| \| ADRA2A \| \| DCP1A \| \| SETD7 \| \| CCNY \| \| REEP1 \| \| HIPK2 \| \| ST18 \| \| KMT2E \| \| TRIM2 \| \| FRS2 \| \| RFX3 \| \| MBOAT2 \| \| SREBF1 \| \| ASXL2 \| \| TSC22D2 \| \| SIM2 \| \| MLXIP \| \| PHF12 \| \| SCN8A \| \| CDR2L \| \| SKI \| \| CDK16 \| \| MDM4 \| \| VCAN \| \| ROCK1 \| \| LARP4B \| \| ST3GAL2 \| \| SNRK \| \| NUFIP2 \| \| ZCCHC14 \| \| ATP11C \| \| MAP4K4 \| \| PTBP3 \| \| PJA2 \| \| IMPA2 \| \| RIMBP2 \| \| SEMA7A \| \| EBAG9 \| \| ANKRD13C \| \| TTLL7 \| \| PI4K2A \| \| FAM126B \| \| FOXP1 \| \| PPP1R9A \| \| TFCP2L1 \| \| LRRC4C \| \|  \| \|  \| \|  \| \|  \| \|  \| \|  \| \|  \| |

* Only targets identified by the target prediction tools of both TargetScan ([www.targetscan.org](http://www.targetscan.org))^1^ and miRNA databases miRDB (<http://www.mirdb.org>)^2-3^ are listed.


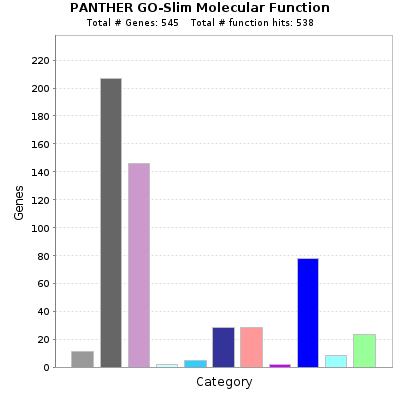

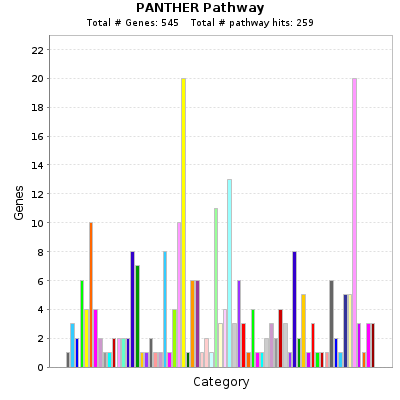


D

A

C

B


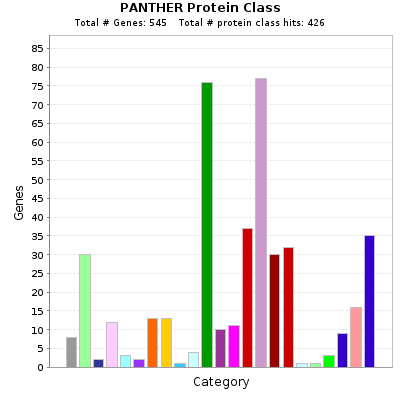

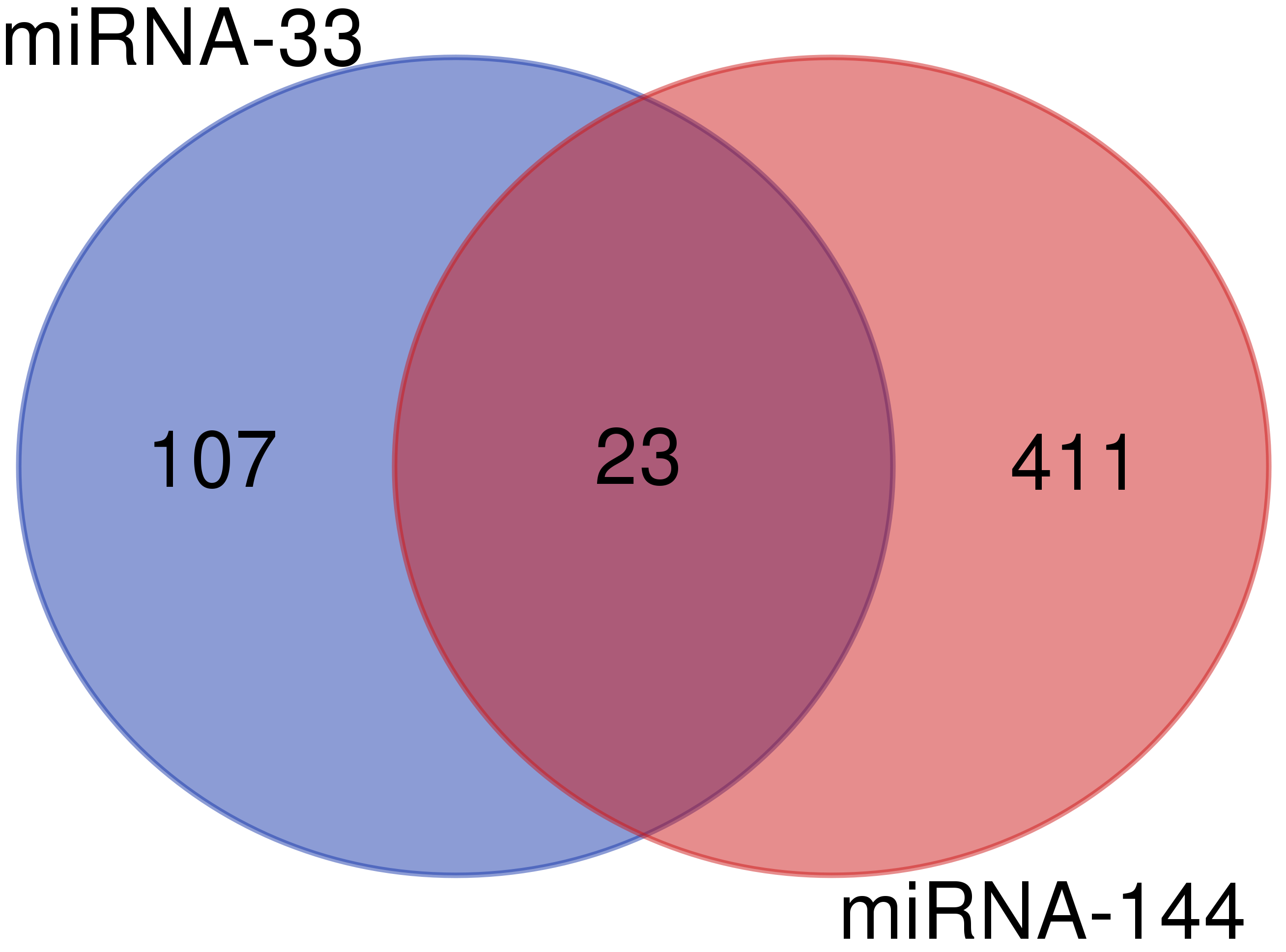


**Chart tooltips are read as: Category name (Accession): # genes; Percent of gene hit against total # genes; Percent of gene hit against total # Function hits

**Chart tooltips are read as: Category name (Accession): # genes; Percent of gene hit against total # genes; Percent of gene hit against total # Protein Class hits

**Chart tooltips are read as: Category name (Accession): # genes; Percent of gene hit against total # genes; Percent of gene hit against total # Pathway hits


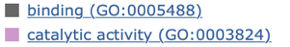

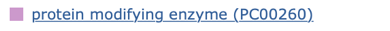

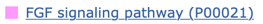


**
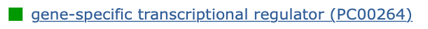
**
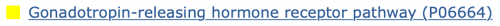

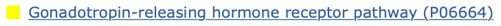


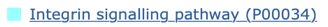


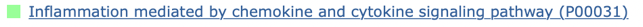

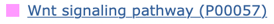


E

F

G

**Figure S4.** **Analysis of miRNA-144-3P and miRNA-33-5P targets.** (A) Venn diagram representing common and unique target genes of miRNA-144-3P and miRNA-33-5P (<http://bioinformatics.psb.ugent.be/webtools/Venn>). Gene Ontology (GO) based classification of miRNA-144-3p and miRNA-33-5p target genes. A total of 545 predicted miRNA-target genes were functionally classified into various categories according to molecular functions (B), pathway (C), and protein class (D), using PANTHER Classification System (<http://pantherdb.org/>)^4^**.** Convergence of miRNA-144-3p and miRNA-33-5p target genes with respect to their molecular function (E), pathway (F), and protein class (G), as a possible mechanism for the synergistic effects found after inhibition of both miRNAs.

C

B

**
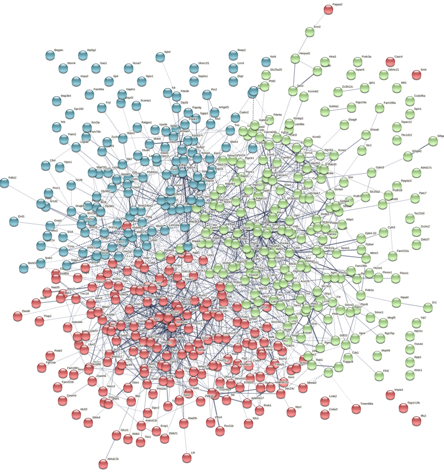
**
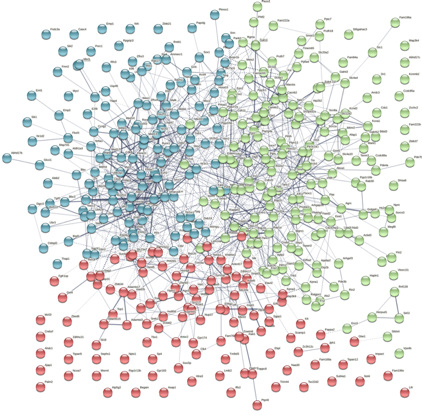


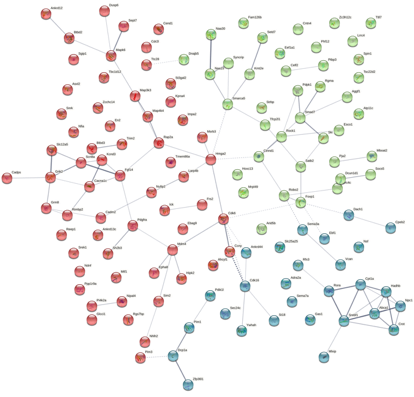


A

**Figure S5.** **Cluster analysis of protein-protein interaction (PPI) network.** (A) 433 genes in PPI network of predicted target genes of miRNA-144:
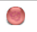
cluster 1: phosphorylation and splicing
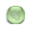
cluster 2: gene expression,
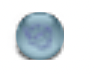
cluster 3: signal transduction. (B) 130 genes in PPI network of predicted target genes of miRNA-33. No clusters. (C) 540 genes in PPI network of predicted target genes of miRNA-144 and miRNA-33 combined:
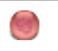
cluster 1: signal transduction, TGF signaling pathway, miRNA,
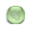
cluster 2: gene expression,
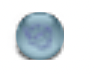
 cluster 3: membrane trafficking ([www.string-db.org](http://www.string-db.org) (version 11.5)^5^.


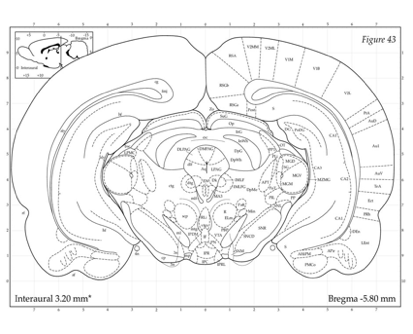

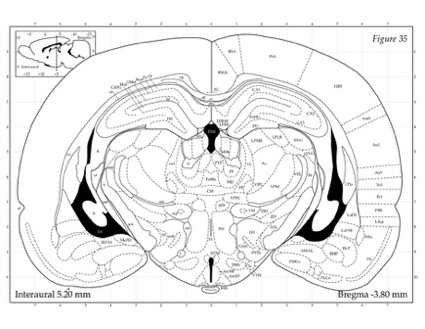


B

A


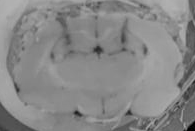

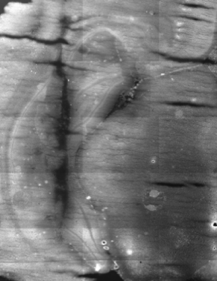


C


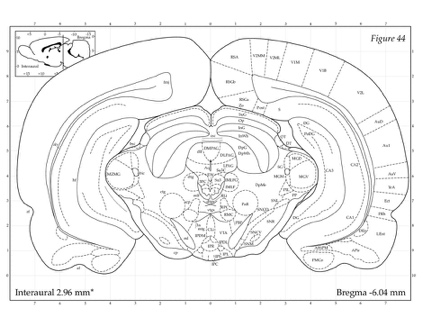

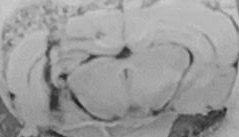


**Figure S6.** **Schematic representation of the cannula placement.** The left section in each image demonstrates cannula tracer in the dorsal DG (A) and ventral DG of male rat (B) and ventral CA1 of female rat. The right sections indicate the specific location of the hippocampal region using Rat Brain Atlas.

**Table S2: Additional statistical results**

| **Fig** | **Hippocampal sub-region** | **Statical test** | **Result** |
| --- | --- | --- | --- |
| **Male** |  | | |
| **miRNA-144** |  | | |
| 3A | dDG | Kruskal-Wallis,  post hoc pairwise comparison | **Type of stress exposure:** H(3)=6.87, n.s |
| 3B | vDG | Kruskal-Wallis,  post hoc pairwise comparison | **Type of stress exposure:** H(3)=5.29, n.s |
| 3C | dCA1 | Kruskal-Wallis,  post hoc pairwise comparison | **Type of stress exposure:** H(3)=0.22, n.s  **Behavioral profile:** H(2)=0.54, n.s |
| 3D | vCA1 | Kruskal-Wallis,  post hoc pairwise comparison | **Type of stress exposure:** H(3)=1.49, n.s  **Behavioral profile:** H(2)=1.41, n.s |
| **miRNA-33** |  | | |
| 3E | dDG | Tow-way ANOVA, post hoc Fisher's LSD | **Type of stress exposure:** F(2,82)=1.74, n.s  **Behavioral profile:** F(1,82)=0.004, n.s  **Type of stress exposure x behavioral profile:** F(2,82)=0.20, n.s |
| 3F | vDG | Tow-way ANOVA, post hoc Fisher's LSD | **Type of stress exposure:** F(2,81)=0.03, n.s  **Type of stress exposure x behavioral profile:** F(2,81)=1.45, n.s |
| 3G | dCA1 | Kruskal-Wallis,  post hoc pairwise comparison | **Type of stress exposure:** H(3)=4.4, n.s  **Behavioral profile:** H(2)=4.02, n.s |
| 3H | vCA1 | Kruskal-Wallis,  post hoc pairwise comparison | **Type of stress exposure:** H(3)=4.22, n.s  **Behavioral profile:** H(2)=4.57, n.s |
| **miRNA-381** |  | | |
| S3A | dDG | Tow-way ANOVA, post hoc Fisher's LSD | **Type of stress exposure:** F(2,82)=0.32, n.s  **Behavioral profile:** F(1,82)=1.28, n.s  **Type of stress exposure x behavioral profile:** F(2,82)=0.07, n.s |
| S3B | vDG | Tow-way ANOVA, post hoc Fisher's LSD | **Type of stress exposure:** F(2,81)=0.29, n.s  **Behavioral profile:** F(1,81)=2.09, n.s  **Type of stress exposure x behavioral profile:** F(2,81)=0.89, n.s |
| S3C | dCA1 | Tow-way ANOVA, post hoc Fisher's LSD | **Type of stress exposure:** F(2,76)=0.32, n.s  **Behavioral profile:** F(1,76)=0.05, n.s  **Type of stress exposure x behavioral profile:** F(2,76)=0.76, n.s |
| S3D | vCA1 | Tow-way ANOVA, post hoc Fisher's LSD | **Behavioral profile:** F(1,74)=3.36, n.s  **Type of stress exposure x behavioral profile:** F(2,74)=0.23, n.s |
| **Female** |  | | |
| **miRNA-144** |  | | |
| 4A | dDG | Kruskal-Wallis,  post hoc pairwise comparison | **Type of stress exposure:** H(3)=4.01, n.s  **Behavioral profile:** H(2)=1.17, n.s |
| 4B | vDG | Tow-way ANOVA, post hoc Fisher's LSD | **Type of stress exposure:** F(2,68)=0.56, n.s  **Behavioral profile:** F(1,68)=0.1, n.s  **Type of stress exposure x behavioral profile:** F(2,68)=0.38, n.s |
| 4C | dCA1 | Kruskal-Wallis,  post hoc pairwise comparison | **Type of stress exposure:** H(3)=1.32, n.s  **Behavioral profile:** H(2)=1.27, n.s |
| 4D | vCA1 | Tow-way ANOVA, post hoc Fisher's LSD | **Type of stress exposure:** F(2,66)=0.08, n.s  **Type of stress exposure x behavioral profile:** F(2,66)=1.17, n.s |
| **miRNA-33** |  | | |
| 4E | dDG | Kruskal-Wallis,  post hoc pairwise comparison | **Type of stress exposure:** H(3)=6.38, n.s  **Behavioral profile:** H(2)=1.73, n.s |
| 4F | vDG | Kruskal-Wallis,  post hoc pairwise comparison | **Type of stress exposure:** H(3)=2, n.s  **Behavioral profile:** H(2)=0.54, n.s |
| 4G | dCA1 | Kruskal-Wallis,  post hoc pairwise comparison | **Type of stress exposure:** H(3)=6.32, n.s  **Behavioral Profile:** H(2)=0.54, n.s |
| 4H | vCA1 | Tow-way ANOVA, post hoc Fisher's LSD | **Type of stress exposure:** F(2,65)=1.13, n.s  **Type of stress exposure x behavioral profile:** F(2,65)=1.7, n.s |
| **miRNA-381** |  | | |
| S3E | dDG | Kruskal-Wallis,  post hoc pairwise comparison | **Type of stress exposure:** H(3)=0.33, n.s  **Behavioral profile:** H(2)=0.84, n.s |
| S3F | vDG | Tow-way ANOVA, post hoc Fisher's LSD | **Type of stress exposure:** F(2,68)=0.23, n.s  **Behavioral profile:** F(1,68)=3.58, n.s  **Type of stress exposure x behavioral profile:** F(2,68)=1.58, n.s |
| S3G | dCA1 | Tow-way ANOVA, post hoc Fisher's LSD | **Type of stress exposure:** F(2,64)=0.53, n.s  **Behavioral profile:** F(1,64)=0, n.s  **Type of stress exposure x behavioral profile:** F(2,64)=0.64, n.s |
| S3H | vCA1 | Tow-way ANOVA, post hoc Fisher's LSD | **Behavioral profile:** F(1,67)=2.05, n.s  **Type of stress exposure x behavioral profile:** F(2,67)=5.10, n.s |

**References**

1. Agarwal, V., Bell, G. W., Nam, J. W., & Bartel, D. P. Predicting effective microRNA target sites in mammalian mRNAs. *Elife* 2015; 4: e05005.‏

2. Wong, N., & Wang, X. miRDB: an online resource for microRNA target prediction and functional annotations. *Nucleic acids research* 2015*;* 43: D146-D152.‏

3. Chen, Y., & Wang, X. miRDB: an online database for prediction of functional microRNA targets. *Nucleic acids research* 2020*;* 48: D127-D131.‏

4. Mi, H., Ebert, D., Muruganujan, A., Mills, C., Albou, L. P., Mushayamaha, T., & Thomas, P. D. PANTHER version 16: a revised family classification, tree-based classification tool, enhancer regions and extensive API. *Nucleic acids research* 2021*;* 49: D394-D403.‏

5. Mering, C. V., Huynen, M., Jaeggi, D., Schmidt, S., Bork, P., & Snel, B. STRING: a database of predicted functional associations between proteins. *Nucleic acids research* 2003*;* 31: 258-261.‏
